# PKCα diffusion and translocation are independent of an intact cytoskeleton

**DOI:** 10.1038/s41598-017-00560-7

**Published:** 2017-03-28

**Authors:** Xin Hui, Benjamin Sauer, Lars Kaestner, Karsten Kruse, Peter Lipp

**Affiliations:** 1 Institute for Molecular Cell Biology, Research Centre for Molecular Imaging and Screening, Center for Molecular Signaling (PZMS), Medical Faculty, Saarland University, Homburg/Saar, Germany; 2 NCCR Chemical Biology, Departments of Biochemistry and Theoretical Physics, University of Geneva, 30 quai Ernest-Ansermet, 1211 Geneva, Switzerland

## Abstract

Translocation of cytosolic cPKC to the plasma membrane is a key event in their activation process but its exact nature is still unclear with particular dispute whether sole diffusion or additional active transport along the cell’s cytoskeleton contributes to cPKC’s dynamics. This was addressed by analyzing the recruitment behavior of PKCα while manipulating the cytoskeleton. Photolytic Ca^2+^ uncaging allowed us to quantify the kinetics of PKCα redistribution to the plasma membrane when fused to monomeric, dimeric and tetrameric fluorescence proteins. Results indicated that translocation kinetics were modulated by the state of oligomerization as expected for varying Stokes’ radii of the participating proteins. Following depolymerization of the microtubules and the actin filaments we found that Ca^2+^ induced membrane accumulation of PKCα was independent of the filamentous state of the cytoskeleton. Fusion of PKCα to the photo-convertible fluorescent protein Dendra2 enabled the investigation of PKCα-cytoskeleton interactions under resting conditions. Redistribution following spatially restricted photoconversion showed that the mobility of the fusion protein was independent of the state of the cytoskeleton. Our data demonstrated that in living cells neither actin filaments nor microtubules contribute to PKCα’s cytosolic mobility or Ca^2+^-induced translocation to the plasma membrane. Instead translocation is a solely diffusion-driven process.

## Introduction

As a member of the conventional Protein kinase C (cPKC) subfamily PKCα serves as a critical intracellular signal translator, transferring Ca^2+^ and lipid signals downstream to phosphorylation events in living cells^1, 2^. In general, after maturation and priming PKCα localizes in the cytosol with the pseudosubstrate domain occupying its kinase cavity thus silencing its kinase activity^3, 4^. When intracellular Ca^2+^ increases, two Ca^2+^ ions bind to the C2 domain of PKCα molecule. This dramatically changes the affinity of the C2 domain to the inner leaflet of the plasma membrane from a repelling state in rest to an attraction state, resulting in the translocation of PKCα protein from the cytosol to the plasma membrane^5–7^. Upon C1 domain mediated binding to diacylglycerol (DAG) the configuration of PKCα changes substantially, leading to an extraction of the pseudosubstrate domain from its kinase catalytic core, relief from inhibition, and initiation of the kinase activity of PKCα^8, 9^.

This highlights the central role of translocation to the plasma membrane for PKCα activation. It has been reported that Ca^2+^-C2 domain binding and protein-membrane association are very transient *in vitro* ^10, 11^. In line with that, we have reported a very fast association and dissociation process of PKCα to/from the plasma membrane by UV-flash photolysis of caged-Ca^2+^ and a caged-Ca^2+^ buffer, respectively^7^. This indicates that Ca^2+^ unbinding and cPKC membrane dissociation are very fast^2^. We and others have shown previously that PKCα translocation from the cytosol to the plasma membrane readily follows intracellular Ca^2+^ oscillations^7, 12–14^. This raises the question how the PKCα proteins translocate from the cytosol to the plasma membrane during such short time periods. Michael Schaefer and colleagues revealed the existence of a transient subplasmalemmal depletion zone of PKCα during its Ca^2+^-induced plasma membrane accumulation. They interpreted this finding in favor of a diffusion-limited distribution process instead of active transport^15^. Primarily only in the proximity of the membrane the rapid PKCα association with the inner leaflet of the plasma membrane via the Ca^2+^ bridge may result in a directional movement that initially depletes the subplasmalemmal cytosol, generating a gradient from the subplasmalemmal space towards the perinuclear cytosol. Later, diffusion equilibrates this gradient, which results in directed cPKC translocation from the cytosol to the plasma membrane. Such a notion, however, still mainly relies on computational simulations and is lacking direct experimental evidence.

In addition, there is a substantial body of evidence that discusses the direct involvement of the cytoskeleton in this dynamic translocation process. Apart from scaffolding tasks cytoskeletal filaments such as actin filaments and microtubules are engaged in a variety of intracellular transport processes, signal transduction, and cell movements^16, 17^. In multiple types of mammalian cells it has been reported that cytoskeletal components are either associated to PKCα^18, 19^ or might be involved in PKCα activation^20^. Moreover, active PKCα might also modulate cytoskeleton structure^21–23^. These reports suggest a rather intimate relationship between an intact cytoskeleton and PKCα.

Here we employed high speed life cell confocal microscopy to investigate the process of PKCα redistribution under resting conditions and during Ca^2+^ mediated translocation in the absence and presence of intact actin filaments and microtubules.

## Results

### Quantitative characterization of Ca^2+^-induced PKCα translocation

**Figure 1:**
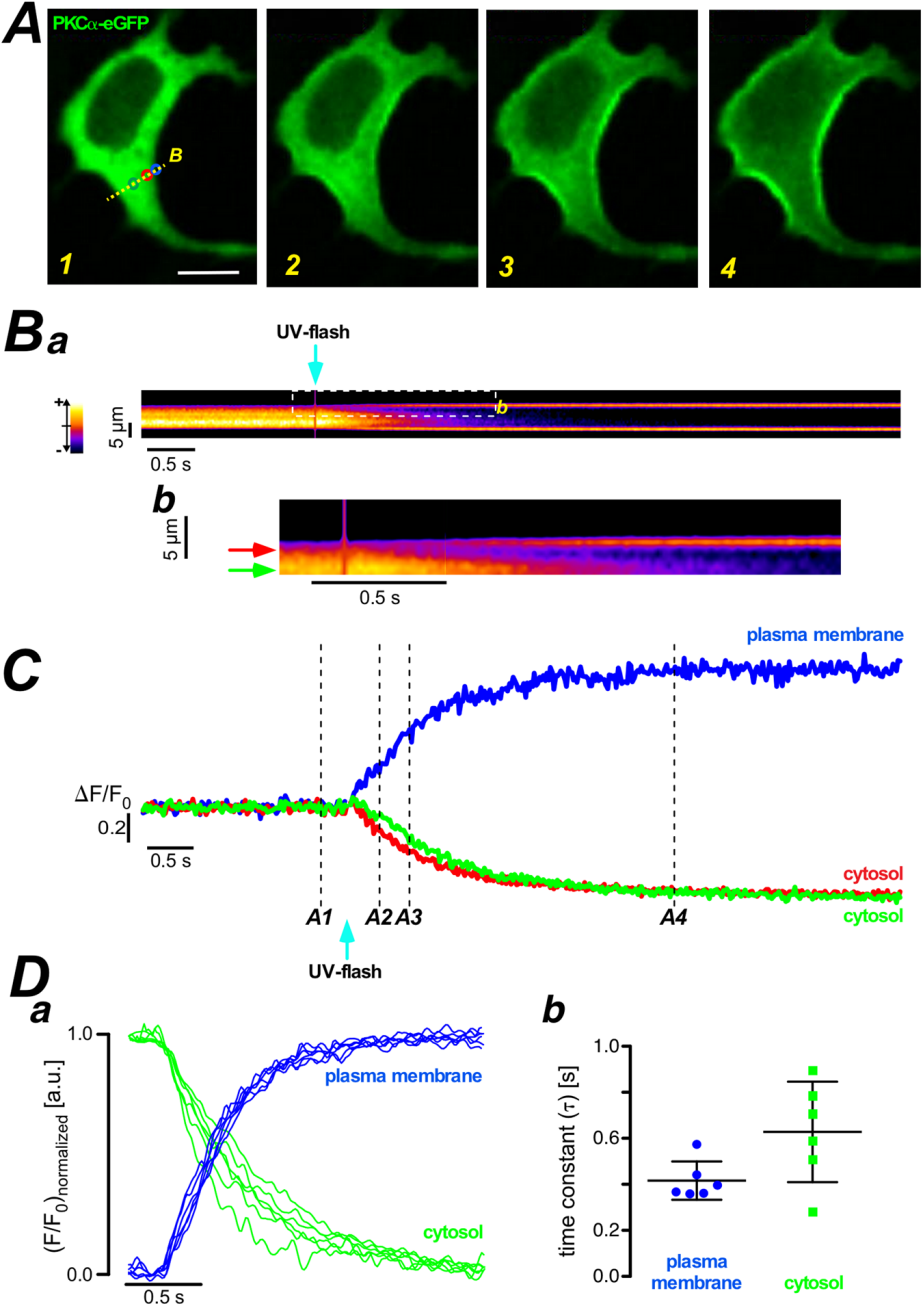
Quantitative analysis of PKCα-eGFP translocation from cytosol to plasma membrane. (**A**) PKCα-eGFP distribution in HEK293 cells at the resting state – left panel. Fluorescent images of PKCα-eGFP distribution upon UV-flash in NP-EGTA loaded HEK293 cells at the time points indicated in (**C**). Scale bar is 10 μm. (Ba) The pseudo line scan at the labeled position (yellow dash line in the panel A1). Part of the pseudo line scan image (dashed box) was redrawn at a higher magnification in (Bb). The color wedge in (Ba) shows the color-coding of the relative fluorescence changes as indicated. (**C**) Plots of fluorescence over time at the plasma membrane (blue) and the cytosol (green and red) from the regions of interest, marked in panel A1. The numbers at the traces correspond to the numbered images in (**A**). (**Da**) Fluorescence over time plots of 6 regions of interest randomly chosen in the cytosolic (green) and at the plasma membrane (blue). (Db) Statistical summary of the analysis of the time constant for the traces depicted in the left panel. The scatter plot depicts individual values (symbols), the mean and the standard deviation. Similar results were found in all 7 cells analyzed in a similar way.

A key step in the activation of cPKCs is the Ca^2+^ dependent translocation from the cytosol to the plasma membrane^1, 2^. To characterize this translocation process kinetically we loaded PKCα-eGFP expressing HEK293 cells with a caged Ca^2+^ compound (NP-EGTA) and rapidly photo-released Ca^2+^ by application of a short high-energy UV-flash. While this maneuver resulted in a quasi-instantaneous increase of the cytosolic Ca^2+^ concentration (Fig. S1), the translocation of PKCα-eGFP was substantially slower and took place over the time course of a couple of seconds (Figs 1A–C, S1). A detailed analysis of pseudo line scans (Fig. 1B) created from 2D image sequences revealed that the cytosolic loss of PKCα-fluorescence was more rapid just beneath the plasma membrane (red arrow in Fig. 1Bb and red trace in Fig. 1C) when compared to deeper layers of the cytosol (green arrow in Fig. 1Bb and green trace in Fig. 1C) resembling the early subplasmalemmal concentration trough observed by Schaefer and co-workers during activation of G-protein coupled signaling cascades^15^. We therefore speculated that the kinetic analysis of the cytosolic fluorescence loss might display a much larger variability depending on where exactly the signal was analyzed when compared to the fluorescence accumulation on the plasma membrane. For this we randomly chose regions of interest on the plasma membrane (Fig. 1D, blue traces and dots) or in the cytosol (Fig. 1D, green traces and squares) and characterized their kinetic changes by fitting mono-exponential decays or upstrokes, with characteristic times τ, to the cytosolic and plasma membrane time courses, respectively. The scatter plot in Fig. 1Db clearly depicts that the spread of time constants from cytosolic regions was substantially larger than that from regions on the plasma membrane; with an SD of 0.22 and 0.08, respectively. A similar distribution was found in all cells analyzed in this way (7 cells). We therefore concluded that analysis of plasma membrane fluorescence accumulation was more reliable and was thus chosen in the following.

### Translocation properties of PKCα with various degrees of oligomerization

**Figure 2:**
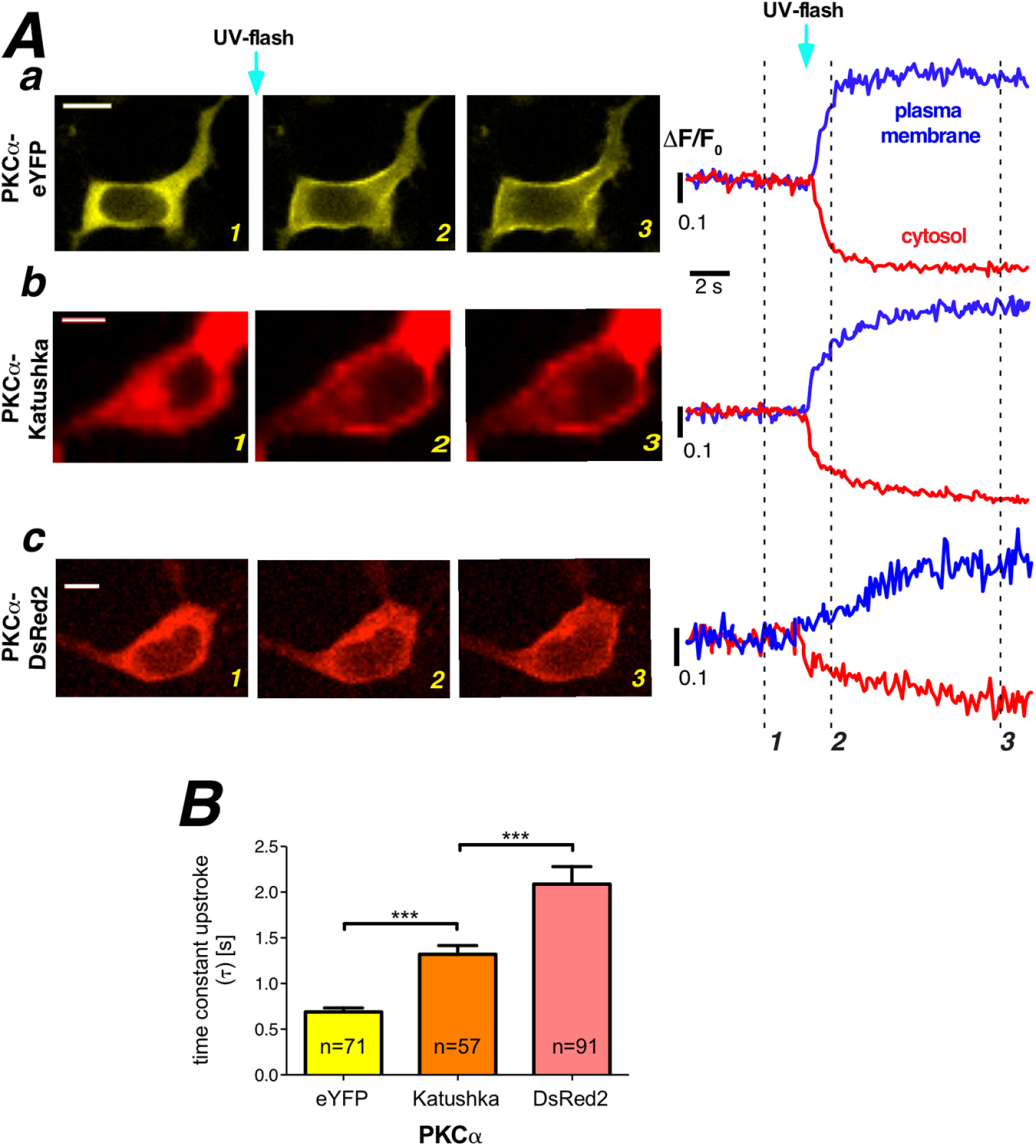
Properties of oligomerized PKCα-fluorescent protein. (**A**) Properties of PKCα translocation to the plasma membrane following UV flash photolysis for (Aa) PKCα-eYFP (monomeric fluorescent protein), (Ab) PKCα-Katushka (dimeric fluorescent protein), and (Ac) PKCα-DsRed2 (tetrameric fluorescent protein). Labeling of the images corresponds to the three different time points highlighted with the dashed lines. Scale bars indicate 10 μm. (**B**) Translocation processes were quantified by fitting a mono-exponential function to the plasma membrane association traces. Statistical summary for PKCα-eYFP, PKCα-Katushka, and PKCα-DsRed2. Numbers given on the bars indicate number of cells in at least 5 independent experiments. Scale bars indicate 10 μm.

To verify and investigate the sensitivity of our approach in characterizing the Ca^2+^ dependent translocation kinetics of PKCα we utilized the availability of fluorescent proteins as monomers (e.g. eYFP, mYFP), dimers (e.g. Katushka) and tetramers (e.g. DsRed2)^24–26^. Such PKCα fusion proteins were expected to reveal substantially altered translocation behavior, independent of whether active transport processes do contribute to the translocation process. HEK293 cells expressing either of the PKCα fusion proteins were loaded with NP-EGTA and protein translocation was characterized following a UV-flash induced Ca^2+^ increase by fitting the plasma membrane fluorescence upstroke to an mono-exponential equation. Typical results for eYFP, Katushka and DsRed2, are displayed in Fig. 2Aa–c, respectively (for comparison of the eYFP used here and mYFP we refer to Fig. S2Aa,B). The statistical analysis is summarized in Fig. 2B depicting the expected slowing down of the translocation process from eYFP over Katushka to DsRed2. In an attempt to quantify the correlation between the oligomerization and the time constant of plasma membrane accumulation we calculated the Pearson correlation coefficient to be 0.9913 indicating a strong correlation between the two parameters. These findings strongly indicated that our approach of characterizing Ca^2+^ dependent translocation of PKCα was sensitive and also suggested diffusion to be a major contributor to this redistribution process.

### Ca^2+^-induced PKCα translocation is independent of intact actin filaments and microtubules

**Figure 3:**
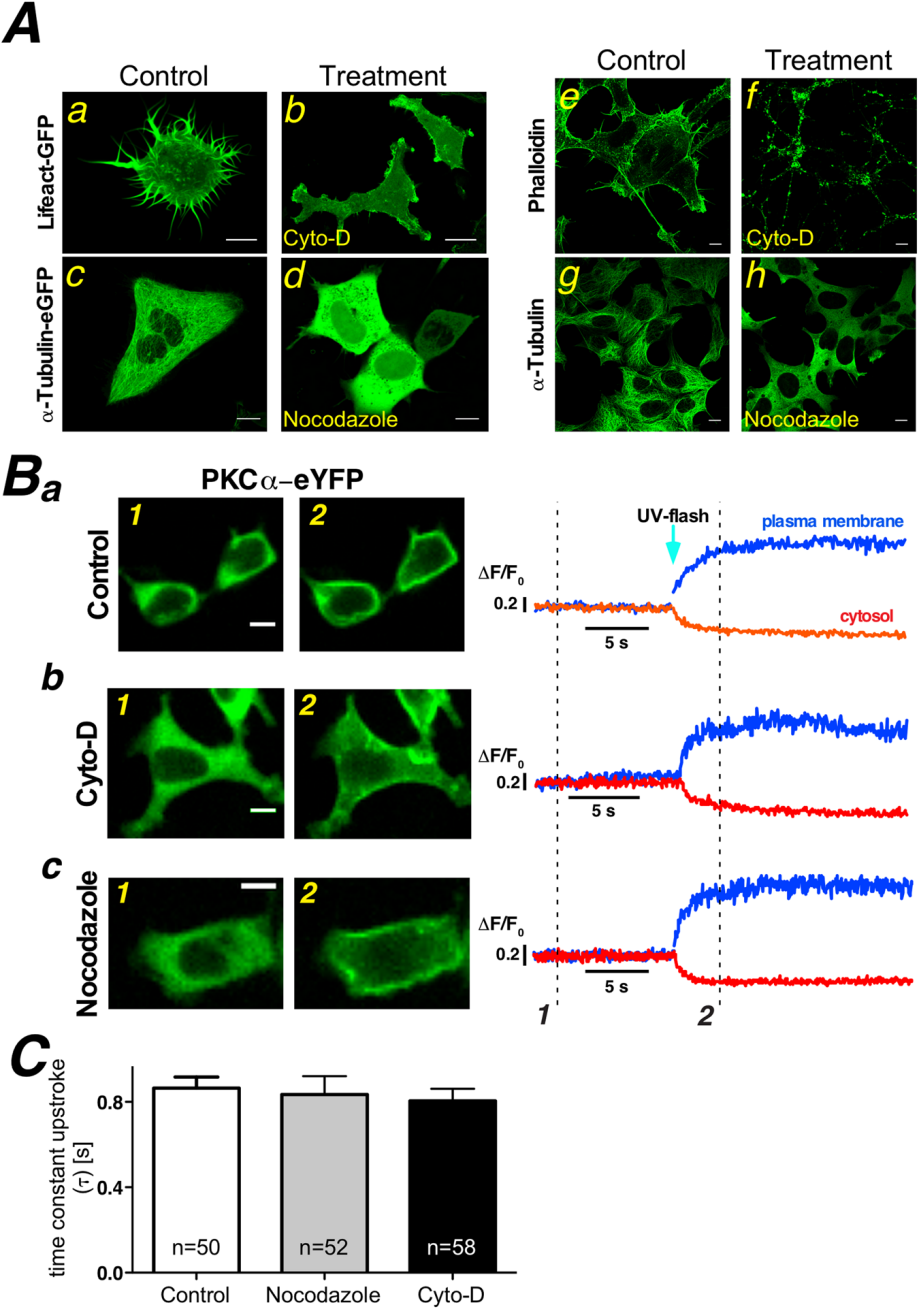
Depolymerization of actin filaments and microtubules does not alter Ca^2+^ induced PKCα translocation. (**A**) Cytochalasin-D (4 µM, 2 hours) (b,f) or nocodazole (10 µM, 2 hours) (d,h) treatment of HEK cells, either expressing Lifeact-GFP (a,b) and α-tubulin-eGFP (c,d), staining with phalloidin (e,f) or immunofluorescence with primary antibodies against α-tubulin (g,h), results in depolymerisation of actin filaments (b,f) and microtubules (d,h), respectively. Note that the confocal sections in Aa and Ab were deliberately close to the bottom of the cell to highlight the spiky plasma membrane protrusion while all other confocal sections were placed in the middle of the nucleus. (**B**) Exemplified single images of PKCα-eYFP distribution before (Ba1, Bb1, Bc1) and following (Ba2, Bb2, Bc2) photolytic Ca^2+^ increase at the time points marked in the traces to the right. Three different experimental conditions are depicted (Ba-control, Bb-cytochalasin-D treatment, Bc- nocodazole treatment). Traces were generated from regions of interest in the cytosol (red traces) and on the plasma membrane (blue traces). (**C**) The plasma membrane accumulation was characterized by fitting an exponential to the upstroke following the flash photolytic Ca^2+^ increase. The statistical summary demonstrates that the state of polymerization of the cytoskeleton does not influence the speed at which PKCα-eYFP accumulates at the plasma membrane after Ca^2+^ UV flash photolysis. Numbers given on the bars indicate number of cells in at least 5 independent experiments.

It is still unclear whether the cytoskeletal components contribute to translocation process of PKCα . To address this, we utilized our Ca^2+^ photolysis assay together with manipulations of the cytoskeleton. Directional, active transport along the actin cytoskeleton critically depends on the availability of polarized filaments (F-actin) that can be visualized by expressing the actin binding protein Lifeact-GFP^27^ (Fig. 3Aa) or by staining with fluorescently-labeled phalloidin (Fig. 3Ae). Application of cytochalasin-D (Cyto-D), 4 μM for 2 hours, resulted in an almost complete disassembly of filamentous actin structures in all of the cells (compare Fig. 3Aa,e before and Fig. 3Ab,f after treatment). Expression of α-tubulin-eGFP or immunofluorescent staining with an anti-α-tubulin primary antibody allowed the direct visualization of the microtubule network in HEK293 cells staining with fluorescently-labeled phalloidin (Fig. 3Ac,g) and treatment of these cells with nocodazole, 5 μM for 2 hours, resulted in a complete disassembly of microtubules in all cells (Fig. 3Ad,h).

In the following we expressed PKCα-eYFP in HEK293 cells either treated with the extracellular solution in the absence of any drugs (control), nocodazole or Cyto-D and subjected them to the UV photolysis of Ca^2+^ as detailed above. Typical results are depicted in Fig. 3B and the statistical analysis of the plasma membrane accumulation is summarized in Fig. 3C.

**Figure 4:**
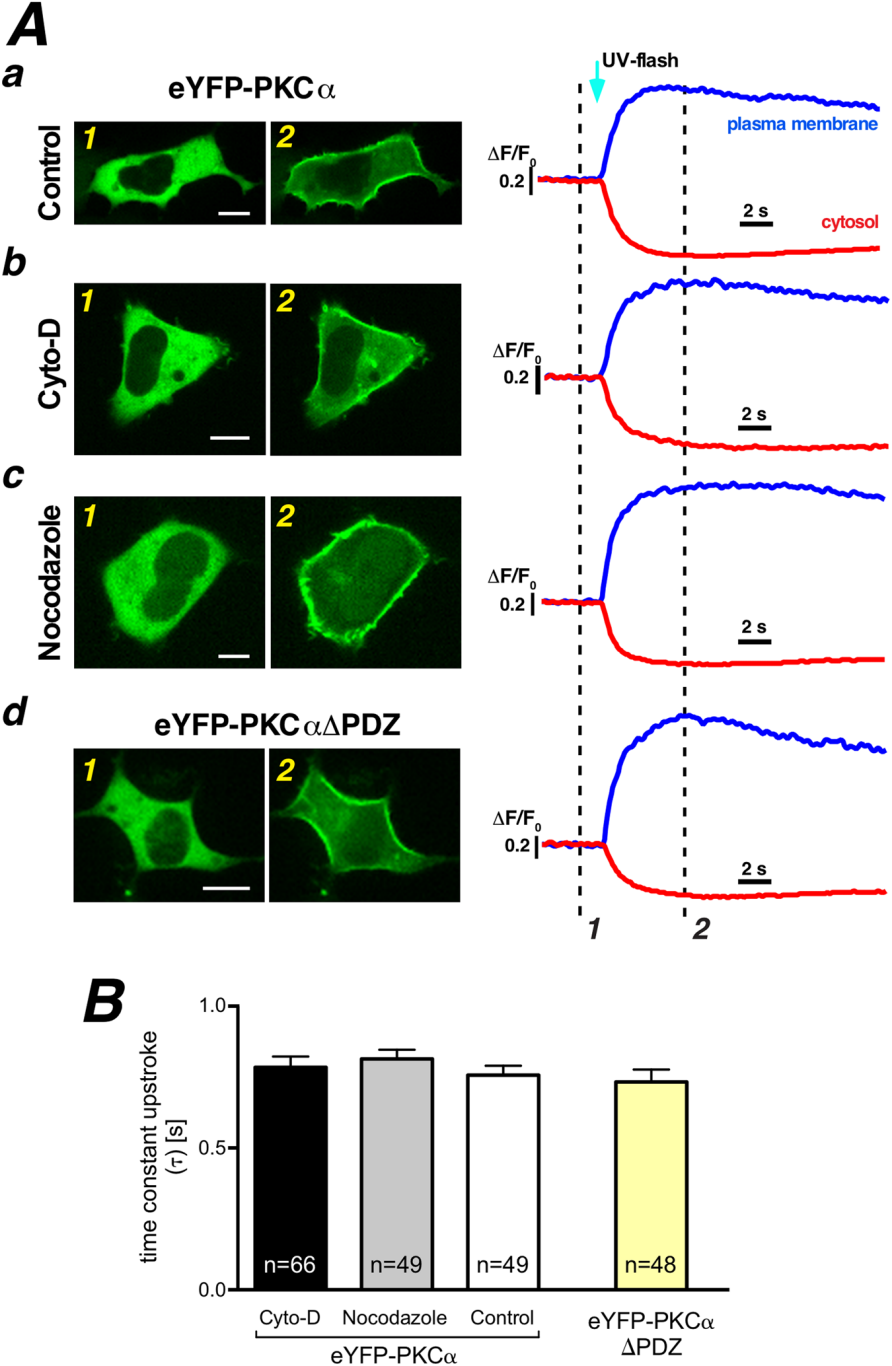
PDZ ligand motif of PKCα does not contribute to Ca^2+^ induced PKCα translocation. (**A**) Exemplified single images of eYFP-PKCα distribution before (Aa1, Ab1, Ac1) and following photolytic Ca^2+^ increase (Aa2, Ab2, Ac2) at the time points marked in the traces to the right. Three different experimental treatments are depicted (Aa-control, Ab-Cyto-D treatment, Ac-nocodazole treatment). Distribution of eYFP-PKCαΔPDZ in HEK293 cell before (Ad1) and after photolytic Ca^2+^ increase (Ad2). Traces were generated from regions of interest in the cytosol (red traces) and on the plasma membrane (blue traces). (**B**) The statistical summary of plasma membrane accumulation time by fitting an exponential to the upstroke following the flash photolytic Ca^2+^ increase. Numbers given on the bars indicate number of cells in at least 5 independent experiments.

The PDZ ligand motif of PKCα locates at the C-terminus of the protein and binds the PDZ domain of scaffold proteins described to influence signal transduction and cell functions^28, 29^. In order to verify whether and to what degree a functional PDZ ligand motif might actually unveil PKCα-cytoskeleton interactions we used a N-terminally fused florescent protein of PKCα, eYFP-PKCα. Interestingly, we observed that the time constant of membrane aggregation of eYFP-PKCα following the UV photolysis of Ca^2+^ was slightly prolonged when compared to that of PKCα-eYFP (Fig. S2Abc,B). To test whether this effect was indeed due to PDZ ligand mediated protein interactions we measured the translocation behavior of a PKCα variant lacking the PDZ-ligand motif, PKCαΔPDZ, but could not detect any difference compared to the full length construct (compare white and yellow bars in Fig. 4B). Together, these results suggested that the slower translocation of eYFP-PKCα as compared to PKCα-eYFP is not due to PDZ-ligand motif mediated protein interactions, but instead might be due to differences in the overall structure of the fusion proteins. For example, those differences could result in slightly altered apparent hydrodynamic radii.

Thereafter, we scrutinized the N-terminal fusion protein (eYFP-PKCα) with an intact PDZ-ligand motif for possible interactions with the cytoskeleton, similar to the approach introduced in Fig. 3. As depicted in Fig. 4, no changes were observed when comparing translocation speeds in the presence (Fig. 4Aa, white bar in B) or the absence of a functional cytoskeleton (Fig. 4Ab,c, black and grey bars in B).

These data indicated that the kinetics of Ca^2+^ triggered PKCα translocation were not altered in the absence of functional cytoskeleton filaments, thus strongly suggesting that active transport along the cytoskeleton is not contributing to the fast, directional Ca^2+^ triggered translocation of PKCα to the plasma membrane.

### In resting cells PKCα’s redistribution behavior is independent of intact actin filaments and microtubules

**Figure 5:**
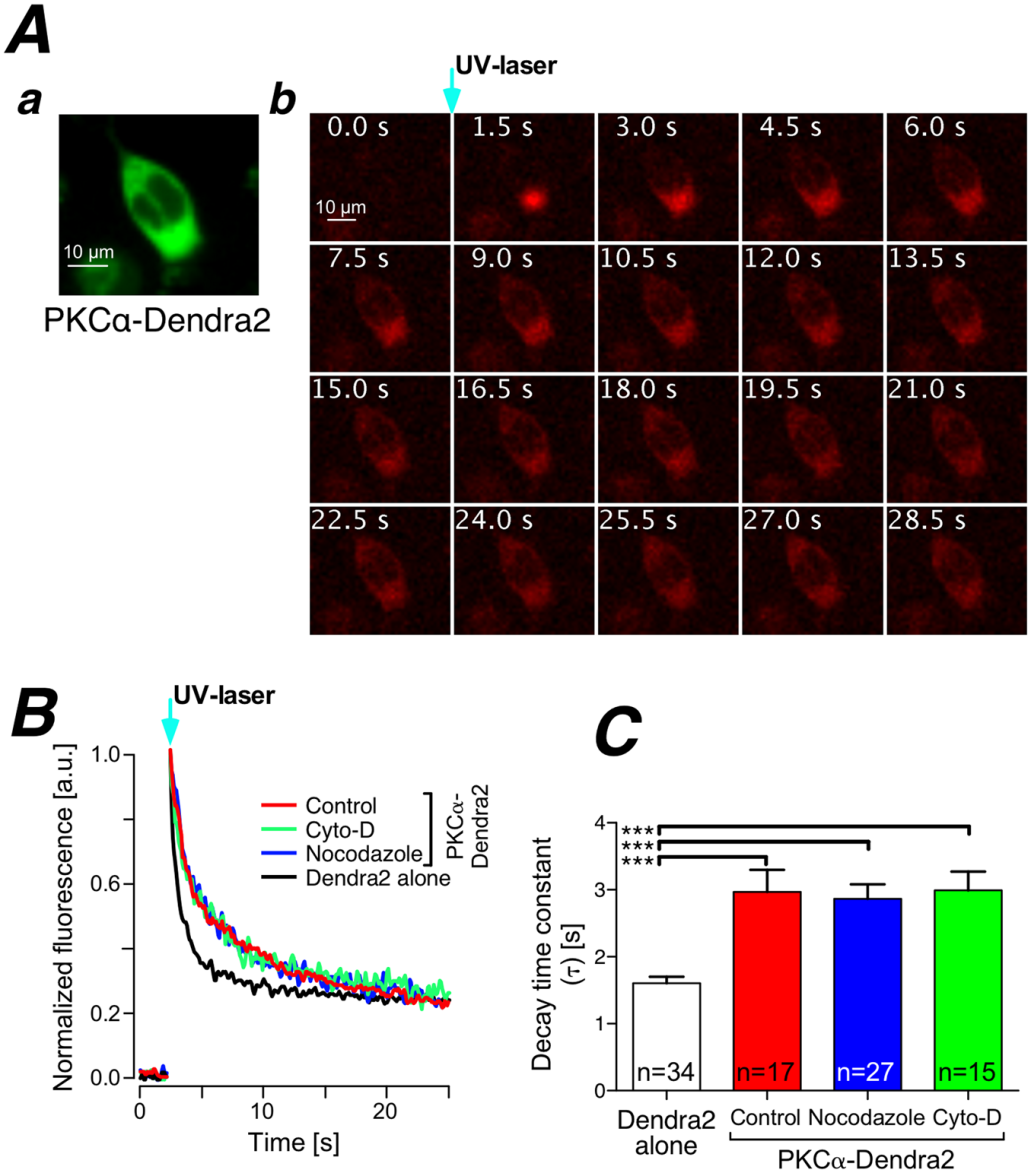
Cytosolic PKCα mobility is not modulated by the state of the cytoskeleton under resting conditions. (**A**) Photoconversion of PKCα-Dendra2 by spatially restricted illumination with a focused UV-laser. Inset shows green fluorescence of Dendra2 under resting conditions. The sequence of images depicts the time course of the red PKCα-Dendra2 fluorescence following UV illumination. Time points are given in each image. (**B**) Fluorescence time course at the point of photoconversion under various experimental conditions (given in the inset). (**C**) Statistical summary of the fluorescence decay at the point of photoconversion during the experimental conditions given. Numbers given on the bars indicate number of cells in at least 3 independent experiments.

So far, our data did not preclude interactions between PKCα and the cytoskeleton under resting, i.e. basal, Ca^2+^ conditions. In order to address this, we made use of the photo-convertible fluorescent protein Dendra2^30^. In its resting state Dendra2 shows green fluorescence that can be switched to red upon photoconversion with blue light. We employed a focused UV laser (spot size around 3 µm in diameter) to cause photoconversion of the PKCα-Dendra2 fusion protein in a spatially restricted manner and followed the diffusion of the photoconverted protein in the presence and absence of a functional cytoskeleton. PKCα-Dendra2 expressed readily in HEK293 cells (Fig. 5Aa) and upon brief photoconversion (300 μs) red fluorescence can be detected that slowly diffused to fill the entire cytoplasm of the cell (see image series in Fig. 5Ab). To characterize the diffusion behavior of the photoconverted PKCα-Dendra2 protein we fitted a mono-exponential function to the fluorescence decay (Fig. 5B). From these data it became evident that Dendra2 alone (black trace in Fig. 5B) expectedly showed the most rapid decay while the PKCα-Dendra2 fusion protein (red trace in Fig. 5B) depicted substantially slower decay kinetics. Interestingly, neither depolymerization of the actin filaments (green trace in Fig. 5B) nor destruction of intact microtubules (blue trace in Fig. 5B) altered that decay rate. Figure 5C summarizes the statistical analysis of a larger population of cells studied and demonstrated that although PKCα-Dendra2’s diffusion was slowed down substantially when compared to Dendra2 alone, all interventions did not alter the diffusion properties of PKCα-Dendra2 highlighting the fact that even under resting conditions, interactions between PKCα and the cytoskeleton did not contribute to the diffusion behavior of this kinase.

## Discussion

Employing flash photolysis of caged Ca^2+^ and fast confocal microscopy in living cells, we established a quantitative method to analyze Ca^2+^-induced translocation of PKCα to the plasma membrane. We found that the different types of oligomerization of PKCα fused fluorescent protein, monomer, dimer, and tetramer slowed down plasma membrane accumulation proportionally indicating that the Ca^2+^ evoked redistribution of PKCα from the cytosol to the subplasmalemmal region is primarily diffusion-driven rather than dependent on active processes. Furthermore, we also revealed that both, Ca^2+^ induced PKCα translocation and PKCα diffusion in the cytosol under rest, are independent of the structural integrity of actin filaments or microtubules in HEK293 cells.

In eukaryotic cells, “simple diffusion”, “facilitated diffusion”, and “active transport” are three main mechanisms for protein transport^31^. “Simple diffusion” is the result of thermal fluctuations of molecules and is often referred to as Brownian motion relying on the thermodynamics of the system. In contrast to “simple diffusion”, “facilitated diffusion” describes a process in which diffusion is sped up by guiding the diffusing particle through association with some other structure. In the case of PKCα it could in principle be that binding to cytoskeletal filaments helps the kinase to navigate through the crowed cytoplasm. In “active transport”, energy is used for moving the molecules which can lead to a directional flow from regions of high concentrations to those of higher concentrations. Taking these thoughts into account for the process of PKCα translocation, “facilitated diffusion” may be the responsible mechanism. Ca^2+^-occupied PKCα molecules rapidly bind to the inner leaflet of the plasma membrane to form nano-clusters as described recently^14^. However, the lifetime of Ca^2+^-PKCα complex is rather brief, around 48 ms^2, 10, 11^ and this taken together with the known diffusion properties of PKCα in the cytosol^15^ the complex can only travel around a micrometer before disassembling again^7^. This short diffusion distance might be the underlying mechanism for the narrow trough of PKCα beneath the plasma membrane (Fig. 1Bb). Together with the membrane association this concentration trough appears to introduce the driving force for “facilitated diffusion”.

Thus, for a given concentration gradient, the speed of protein flux is mostly determined by the hydrodynamic radius of the protein, also called Stokes’ radius, which correlates with the molecular mass of soluble globular proteins^31^. Three forms of oligomerized PKCα-fluorescent proteins, monomer, dimer, and tetramer, were used to address two questions: (i) is the photolysis assay sensitive enough to detect different diffusion rates at all and (ii) is the photolysis assay reliable enough to yield quantitative information about the translocation process. Interestingly, we observed a substantially slower translocation of the PKCα-tetramer when compared to the PKCα-monomer (Fig. 2B), which is compatible to experimental findings with citrine and dsRed^24^. In comparison to the aforementioned report, our situation is more complex because it appears inadequate to describe the PKCα-fluorescent protein (FP) fusion proteins as globular proteins, we would rather refer to them as “two balls on a stick” and thus oligomerization of the FP part will result in rather unpredictable topologies of the resulting protein complex. Moreover, these data not only further reassured a majorly diffusion-driven mechanism as the principal mechanism for PKCα translocation, but also strongly indicated that there is no additional carrier molecule involved in this translocation process.

Beside the hydrodynamic radius, the absolute temperature and the viscosity of the medium are also important determinants in simple diffusion. In living cells, at least during the course of an experiment, the temperature can be seen as constant and is therefore not relevant. The viscosity of the medium, in this case the intracellular cytosol, is one important variable^31^. Using pharmacological approaches, we successfully depolymerized the actin filaments and the microtubules, and analyzed possible effects such interventions might have for the relative speed of PKCα translocation. Because there are no significant differences of the photolytic release of Ca^2+^ (data not shown) and no significant viscosity changes of the cells following Cyto-D or Nocodazole treatment^32–35^, the results from the experiments clearly indicated that neither actin filaments nor microtubules were directly responsible or involved in Ca^2+^-dependent plasma membrane translocation of PKCα. C-terminal fusion proteins do obscure possible PKCα-protein interactions via PKCα’s PDZ-ligand motif and we thus also confirmed that N-terminally fused fluorescent proteins depicted the same properties. Their translocation behaviors were also independent of functional actin filaments or microtubules. Interestingly, the N-terminally fused PKCα variant displayed a significantly slowed down translocation speed. This behavior could either be attributed to a slightly increased hydrodynamic radius of the protein or to a slightly hindered and therefore slower plasma membrane interaction. One should keep in mind that the N-terminus of PKCα contains the C-domains responsible for Ca^2+^- and lipid-dependent membrane interaction.

Nevertheless, one question was remaining: are PKCα molecules associated with the cytoskeleton during their resting state? Cytoskeletal components associated to PKCα have been reported for adrenal cells, melanoma cells^18, 19^, or in cardiomyocytes^20^. Moreover, activated PKCα has the capability to regulate the structure of the cytoskeleton^21–23^. Spatially restricted illumination of photoconvertible fluorescent proteins in living cells provides an ideal approach to study the diffusion process of PKCα in the cytosol and investigate such a dynamic process under the resting condition^31^. As discussed above, the Stokes’ radius of proteins is the key factor determining the diffusion coefficient in living cells. Because photoconversion only changes the fluorescent protein’s inner core in the vicinity of the chromophore, the typical barrel structure will not be altered significantly and thus the Stokes’ radius will remain constant. Considering the value of the Stokes’ radius to be 45.2 Å for YFP fused PKCα and 22.7 Å for free YFP^15^, similar Stokes radii are expected for PKCα-Dendra2 and free Dendra2. The differences in our measured decay time constants for those proteins (Fig. 4C) are consistent with this expectation. Moreover, the diffusion behavior of the fusion protein is not changed following depolymerization of the actin filaments or the microtubules. These data strongly suggested that there is no measureable direct association between PKCα and actin or tubulin molecules. In addition to actin filaments and microtubules, the third component of the cytoskeleton, intermediate filaments, also needs to be discussed. However, because intermediate filaments are not polarized (in contrast to actin filaments and microtubules) directional transport along these filaments appears rather unlikely. Their main function seems to be centered around helping the cell in withstanding mechanical stress rather than mediating transport^16, 36^.

In this study, we applied PKCα-fluorescent protein fusion constructs combined with advanced optical imaging and optical intervention methods in living cells to demonstrate that translocation of PKCα, one member of the cPKC subfamily, appears purely diffusion-driven with no detectable contribution of filamentous transport to the membrane recruitment process, neither along the actin filaments nor along the microtubules. Because protein transport along intermediate filaments also appears rather unlikely, PKCα recruitment to the plasma membrane can be regarded as a purely diffusion-driven process. Nevertheless, changes in the cytoskeletal microarchitecture might indirectly contribute to the diffusion behavior of PKCα on the micrometer scale by either generating inhomogeneity in the apparent viscosity of the cytosol or by actively fencing plasma membrane areas from direct diffusion access from the cytosol.

## Material and Methods

### Cell Culture and Transfection

HEK293 cells were cultured and transfected as previously described^37^. Briefly HEK293 cells were seeded on 20 mm glass coverslips 24 hours before transfection. Using transfection reagent, NanoJuice^®^ (Novagen, USA), plasmids were transfected into HEK293 cells according to the vendor’s recommendations. Cells were investigated 48 hours after transfection.

### Solutions and chemical compounds

All experiments were conducted at room temperature (20–22 °C) in Tyrode’s buffer comprising: 135 mM NaCl, 5.4 mM KCl, 2 mM MgCl_2_, 1.8 mM CaCl_2_, 10 mM glucose, 10 mM HEPES adjusted to pH 7.35 with NaOH. All compounds used were of research grade. Cytochalasin D and Nocodazole were purchased from Sigma (Sigma-Aldrich, Germany).

### Plasmids (Fluorescence-labeled protein)

The wild-type human PKCα protein was fused with eGFP, eYFP or, DsRed2 at the C-terminus in the pCDNA3 plasmid as described previously^7, 38^. The full-length of human PKCα N-terminally fused to eYFP was constructed into the plasmid pEYFP-C by PCR with following primers, 5′ GCCTCGAGTGGCTGACGTTTTCCCGGGC and 5′ GCAGATCT TTATACTGCACTCTGTAAGATGG. The human PKCα without the last three amino acids (PKCα PDZ)^29^ was N-terminally fused to eYFP (into the plasmid pEYFP-C) by PCR with following primers, 5′ GCCTCGAGTGGCTGACGTTTTCCCGGGC and 5′ GCAGATCTTTACTGTAAGATGGGGTGCAC.

The plasmid encoding the monomeric variant of YFP, mYFP, was a gift from Prof. Alexandra Newton^26^. The plasmid encoding the red fluorescent protein Katushka, pTurboFP635-N was a gift from Prof. Dmitriy M. Chudakov^25^. Human PKCα protein fused to Katushka or photoconvertible fluorescent protein Dendra2 (Clontech, USA) was achieved at the C-terminus employing the following primers, 5′ ATCTCGAGGCCCTTGGGACCATGGCT and 5′ AGAATTCCTACTGCACTCTGTAAGATG.

The plasmid encoding Lifeact-GFP was a gift from Prof. Wedlich-Söldner^27^. The plasmid encoding eGFP-α-tubulin was purchased from Clontech (Clontech, USA).

All plasmids were confirmed by sequencing.

### Immunocytofluorescence labeling of actin filaments and microtubules

When HEK293 cells on coverslips reached about 40% confluency, they were fixed in 4% paraformaldehyde (in PBS) for 10 min. After a wash step with PBS, the fixed cells were permeabilized with 0.1% Triton X-100 (in PBS) for 10 min. After a PBS wash, 5% BSA in PBS was used for blocking unspecific binding for 20 min at room temperature. For actin filament staining, 0.5 μM ATTO-647N phalloidin (Atto-Tec, Germany) was employed in 1% BSA of PBS at room temperature for 1 h. Anti-α-tubulin (Sigma-Aldrich, Germany) was employed for microtubules’ staining in 1% BSA of PBS at room temperature for 2 h. The polyclonal secondary antibody (anti mouse from goat; with Alexa Fluor 649 (Jackson ImmunoResearch, USA)) was left on the cells for 1 h (at room temperature). After washing with PBS for 3 times, the cells were mounted in ProLong Gold Antifade reagent and visualized by confocal microscopy.

### Image acquisition

All confocal images from living cells were based on an inverted microscope (TE-2000E, Nikon, Germany) using an oil immersion objective (40x, NA 1.3 S-Fluor, Nikon, Germany). The microscope was attached to a fast 2D-kilobeam array scanner (Infinity-3; VisiTech Int., UK) that simultaneously scans 2500 parallel laser beams across the specimen and projects the resulting fluorescence images on two spectrally separated EMCCD-cameras (iXon 887, Andor Technology, UK). For excitation of eGFP, eYFP, Katushka and DsRed2, we employed respective solid-state lasers; eGFP excitation with a 491 nm laser (Cobolt, Sweden), eYFP excitation with a 514 nm laser (Cobolt, Sweden) and collected fluorescence emission with 515 mm long-pass filter (VisiTech Int., UK). Katushka and DsRed2 were excited with a 561 nm laser (Cobolt, Sweden) and fluorescence emissions were collected with a 570–675 nm band-pass filter (VisiTech Int., UK). The entire setup was integrated and controlled through VoxCellScan software (VisiTech Int., UK).

Experiments using UV flash photolysis of caged Ca^2+^ were performed as described earlier^7^. Briefly, cells were loaded with 7.5 μM NP-EGTA-AM (Invitrogen, Germany) for 30 min. Ca^2+^ was photo-released by a bright UV flash (Rapp OptoElectronic, Germany). Images were recorded at 10 fps with 256 × 256 pixels’ images or at 71 fps with 256 × 30 pixels’ images by Infinity-3 system (VisiTech Int., UK) described above.

Spatially restricted conversion of Dendra2 was achieved by a 379 nm UV laser (Toptica, Germany) focused to a spot of approx. 3 µm in diameter. In order to avoid cross talk of the conversion and imaging, we electronically placed the UV excitation pulse between two successive images (illumination duration 300 μs). Unconverted Dendra2 was illuminated with a 491 nm laser (Cobolt, Sweden) and detected through a 497–550 nm band-pass filter (VisiTech Int., UK) while the photoconverted Dendra2 was detected by illumination with a 561 nm laser (Cobolt, Sweden) and fluorescence detection through a 570–675 nm band-pass filter. Images (128 × 128 pixels) were recorded at 10 fps.

### Data handling

After the experiments, the resulting image series were transferred into a large-scale image database system running OMERO 5.02 (Open microscopy environment, University of Dundee, UK) for long-term storage. Images were processed either in MatLab (see below) or in ImageJ. To obtain fluorescence over time plots, the fluorescence information from regions-of-interest were averaged, saved and imported into Igor software (Wavemetrics, USA).

Where appropriate, we calculated so-called self-ratio traces or images (F/F_0_), for which the fluorescence at any given time point (F) was divided by the resting fluorescence (F_0_) to account for different dye loading and/or expression of the fluorescent proteins and their distribution in subcellular compartments (see also ref. 7).

Final Figure design was performed in Adobe Illustrator CS6 (Adobe, USA) and Canvas Draw 3.0 (ACD, USA).

Statistical analysis of the data was performed in Prism 6 software (GraphPad, USA). After testing for a normal distribution of the data points (D’Agostino-Pearson omnibus normality test) the data were analyzed with an unpaired t-test. Bar graphs depict mean ± SEM unless stated otherwise. Statistical significance was defined as follows; (*)p < 0.05; (**)p < 0.01; (***)p < 0.001. N numbers give the number of cells analyzed, whereas the number of experiments depicts independent experiments (different passage or cover slip).

## Electronic supplementary material

Supplementary Information

## References

[1] Gallegos LL, Newton AC (2008). Spatiotemporal dynamics of lipid signaling: Protein kinase C as a paradigm. IUBMB Life.

[2] Lipp P, Reither G (2011). Protein Kinase C: The ‘Masters’ of Calcium and Lipid. Cold Spring Harb. Perspect. Biol.

[3] House C, Kemp BE (1987). Protein kinase C contains a pseudosubstrate prototope in its regulatory domain. Science.

[4] Steinberg SF (2008). Structural Basis of Protein Kinase C Isoform Function. Physiol. Rev..

[5] Medkova M, Cho W (1998). Mutagenesis of the C2 domain of protein kinase C-alpha. Differential roles of Ca2+ ligands and membrane binding residues. J. Biol. Chem..

[6] Murray D, Honig B (2002). Electrostatic control of the membrane targeting of C2 domains. Mol. Cell.

[7] Reither G, Schaefer M, Lipp P (2006). PKCalpha: a versatile key for decoding the cellular calcium toolkit. J. Cell Biol..

[8] Leonard TA, Różycki B, Saidi LF, Hummer G, Hurley JH (2011). Crystal Structure and Allosteric Activation of Protein Kinase C &beta;II. Cell.

[9] Antal, C. E., Callender, J. A., Kornev, A. P., Taylor, S. S. & Newton, A. C. Intramolecular C2 Domain-Mediated Autoinhibition of Protein Kinase C bII. *Cell Reports* 1–14, doi:10.1016/j.celrep.2015.07.039 (2015). 10.1016/j.celrep.2015.07.039 PMC4551583 26279568

[10] Nalefski EA, Newton AC (2001). Membrane binding kinetics of protein kinase C betaII mediated by the C2 domain. Biochemistry.

[11] Kohout SC, Corbalan-Garcia S, Torrecillas A, Gomez-Fernandez JC, Falke JJ (2002). C2 domains of protein kinase C isoforms alpha, beta, and gamma: activation parameters and calcium stoichiometries of the membrane-bound state. Biochemistry.

[12] Oancea E, Meyer T (1998). Protein kinase C as a molecular machine for decoding calcium and diacylglycerol signals. Cell.

[13] Lipp P, Hui X, Reither G, Kaestner L (2014). Multi-channel imaging of cellular signaling: interplay of Ca2+ and conventional protein kinase C. Cold Spring Harb Protoc.

[14] Bonny M (2016). C2-domain mediated nano-cluster formation increases calcium signaling efficiency. Sci Rep.

[15] Schaefer M, Albrecht N, Hofmann T, Gudermann T, Schultz G (2001). Diffusion-limited translocation mechanism of protein kinase C isotypes. FASEB J.

[16] Doherty GJ, McMahon HT (2008). Mediation, modulation, and consequences of membrane-cytoskeleton interactions. Annu. Rev. Biophys.

[17] He H-T, Marguet D (2011). Detecting Nanodomains in Living Cell Membrane by Fluorescence Correlation Spectroscopy. Annu Rev Phys Chem.

[18] Papadopoulos V, Hall PF (1989). Isolation and characterization of protein kinase C from Y-1 adrenal cell cytoskeleton. J. Cell Biol..

[19] Szalay J (2001). Associations of PKC isoforms with the cytoskeleton of B16F10 melanoma cells. J. Histochem. Cytochem..

[20] Mochly-Rosen D, Henrich CJ, Cheever L, Khaner H, Simpson PC (1990). A protein kinase C isozyme is translocated to cytoskeletal elements on activation. Cell Regul..

[21] Larsson C (2006). Protein kinase C and the regulation of the actin cytoskeleton. Cell Signal.

[22] Evans JH, Falke JJ (2007). Ca2+ influx is an essential component of the positive-feedback loop that maintains leading-edge structure and activity in macrophages. Proc. Natl. Acad. Sci. USA.

[23] Yang Q (2013). Protein kinase C activation decreases peripheral actin network density and increases central nonmuscle myosin II contractility in neuronal growth cones. Mol. Biol. Cell.

[24] Heikal A, Hess S, Baird G, Tsien RY, Webb W (2000). Molecular spectroscopy and dynamics of intrinsically fluorescent proteins: Coral red (dsRed) and yellow (Citrine) (vol 97, pg 11996, 2000). Proc. Natl. Acad. Sci. USA.

[25] Shcherbo D (2007). Bright far-red fluorescent protein for whole-body imaging. Nat. Meth.

[26] Zacharias DA, Violin JD, Newton AC, Tsien RY (2002). Partitioning of lipid-modified monomeric GFPs into membrane microdomains of live cells. Science.

[27] Riedl J (2008). Lifeact: a versatile marker to visualize F-actin. Nat. Meth.

[28] Tsunoda S (1997). A multivalent PDZ-domain protein assembles signalling complexes in a G-protein-coupled cascade. Nature.

[29] O’Neill AK (2011). Protein Kinase C Promotes Cell Migration through a PDZ-Dependent Interaction with its Novel Substrate Discs Large Homolog 1 (DLG1). J. Biol. Chem..

[30] Gurskaya NG (2006). Engineering of a monomeric green-to-red photoactivatable fluorescent protein induced by blue light. Nat. Biotechnol..

[31] Miyawaki A (2011). Proteins on the move: insights gained from fluorescent protein technologies. Nat. Rev. Mol. Cell Biol..

[32] Wang N (1998). Mechanical Interactions Among Cytoskeletal Filaments. Hypertension.

[33] Yanai M (1999). Intracellular elasticity and viscosity in the body, leading, and trailing regions of locomoting neutrophils. Am. J. Physiol..

[34] Wakatsuki T, Schwab B, Thompson N, Elson E (2001). Effects of cytochalasin D and latrunculin B on mechanical properties of cells. J. Cell Sci..

[35] Schulze C, Müller K, Käs JA, Gerdelmann JC (2009). Compaction of cell shape occurs before decrease of elasticity in CHO-K1 cells treated with actin cytoskeleton disrupting drug cytochalasin D. Cell Motil. Cytoskeleton.

[36] Lazarides E (1982). Intermediate filaments: a chemically heterogeneous, developmentally regulated class of proteins. Annu. Rev. Biochem..

[37] Hui X, Reither G, Kaestner L, Lipp P (2014). Targeted Activation of Conventional and Novel Protein Kinases C through Differential Translocation Patterns. Mol. Cell. Biol..

[38] Hui X, Kaestner L, Lipp P (2014). Differential targeting of cPKC and nPKC decodes and regulates Ca2+ and lipid signalling. Biochem. Soc. Trans..

