# Supplementary material for: PKCα diffusion and translocation are independent of an intact cytoskeleton
Source: Sci Rep. 2017 Mar 28;7:475. doi: 10.1038/s41598-017-00560-7 (PMC5428563; doi:10.1038/s41598-017-00560-7)
Supplement: Supplementary file 1 — Supplementary Information [file 41598_2017_560_MOESM1_ESM.pdf]

Title: PKCα diffusion and translocation are independent of an intact cytoskeleton

Authors: Xin Hui[1], Benjamin Sauer[1], Lars Kaestner[1], Karsten Kruse[2] Peter Lipp[1*]

One Sentence Summary: Employing pharmacological and optical tools, we quantitatively demonstrate that the subcellular dynamicity of PKCα is driven by sole diffusion and independent of the integrity of the cytoskeleton.

Affiliations:
[1]Institute for Molecular Cell Biology, Research Centre for Molecular Imaging and Screening, Center for Molecular Signaling (PZMS), Medical Faculty, Saarland University, Homburg/Saar, Germany.

[2] Departments of Biochemistry and Theoretical Physics, University of Geneva, 30 quai Ernest-Ansermet, 1211 Geneva, Switzerland.
*correspondence to peter.lipp@uks.eu

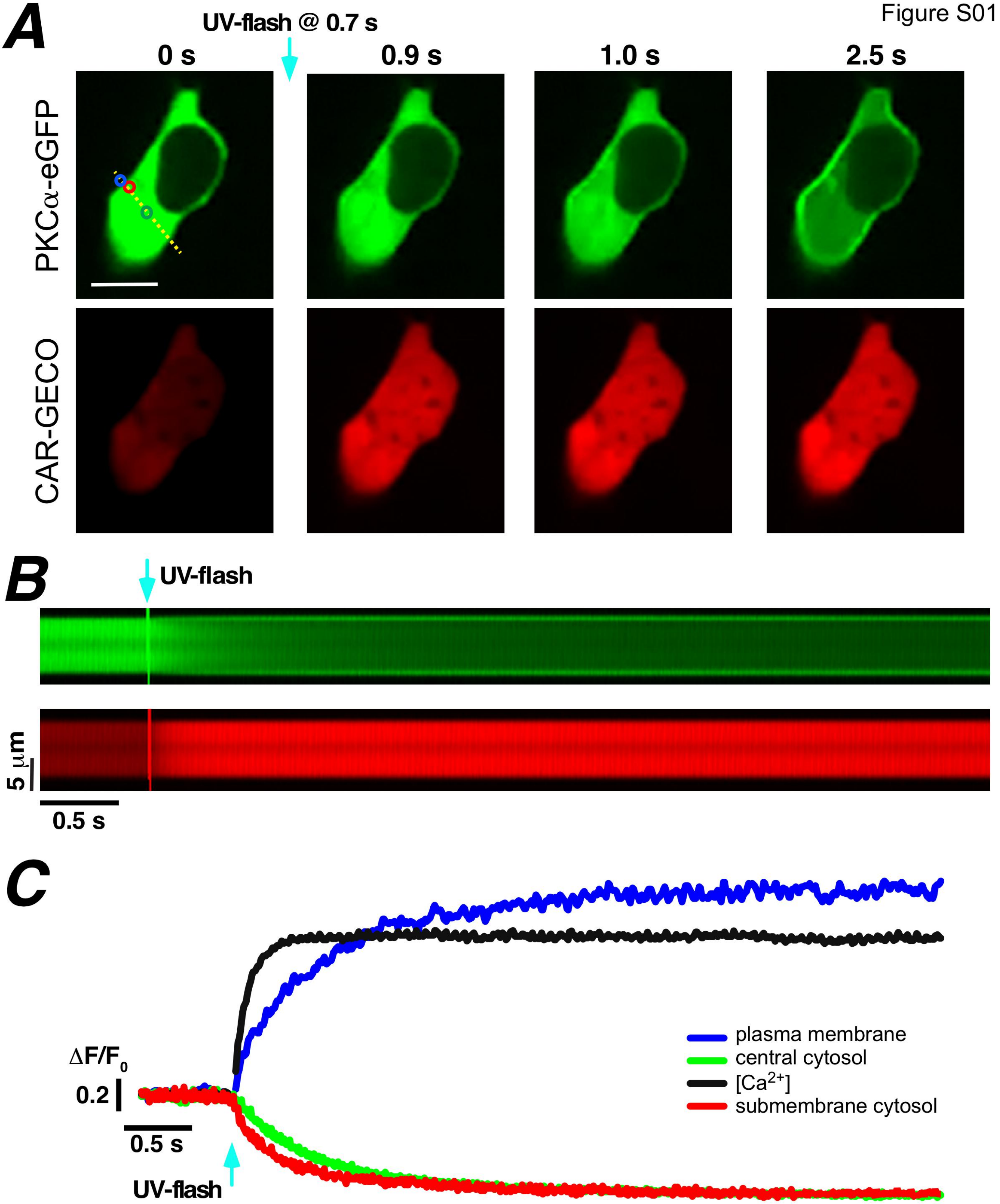

Figure S01. UV-flash evoked NP-EGTA uncaging results in a substantially faster Ca increase than Ca-dependent translocation of PKCα-eGFP. NP-EGTA-AM loaded HEK cells expressing PKCα-eGFP and the red-shifted genetically encoded Ca sensor CAR-GECO were subjected to a birght UV-flash at the time indicated by the turkis arrow. (A) upper row redistribution of PKCα-eGFP and lower row fluorescence changes of the CAR-GECO for the time points given. Scale bar depicts 10 µm. (B) Psudeo-linescan along the yellow dahsed line in (A) shows the redistribution of PKCα-eGFP and the fluorescence change of CAR-GECO. (C) fluorescence over time traces for the subcellular locations given. The flash artefact was removed for display reasons. Similar results were obtained from additional 15 cells from 3 independent experiments.

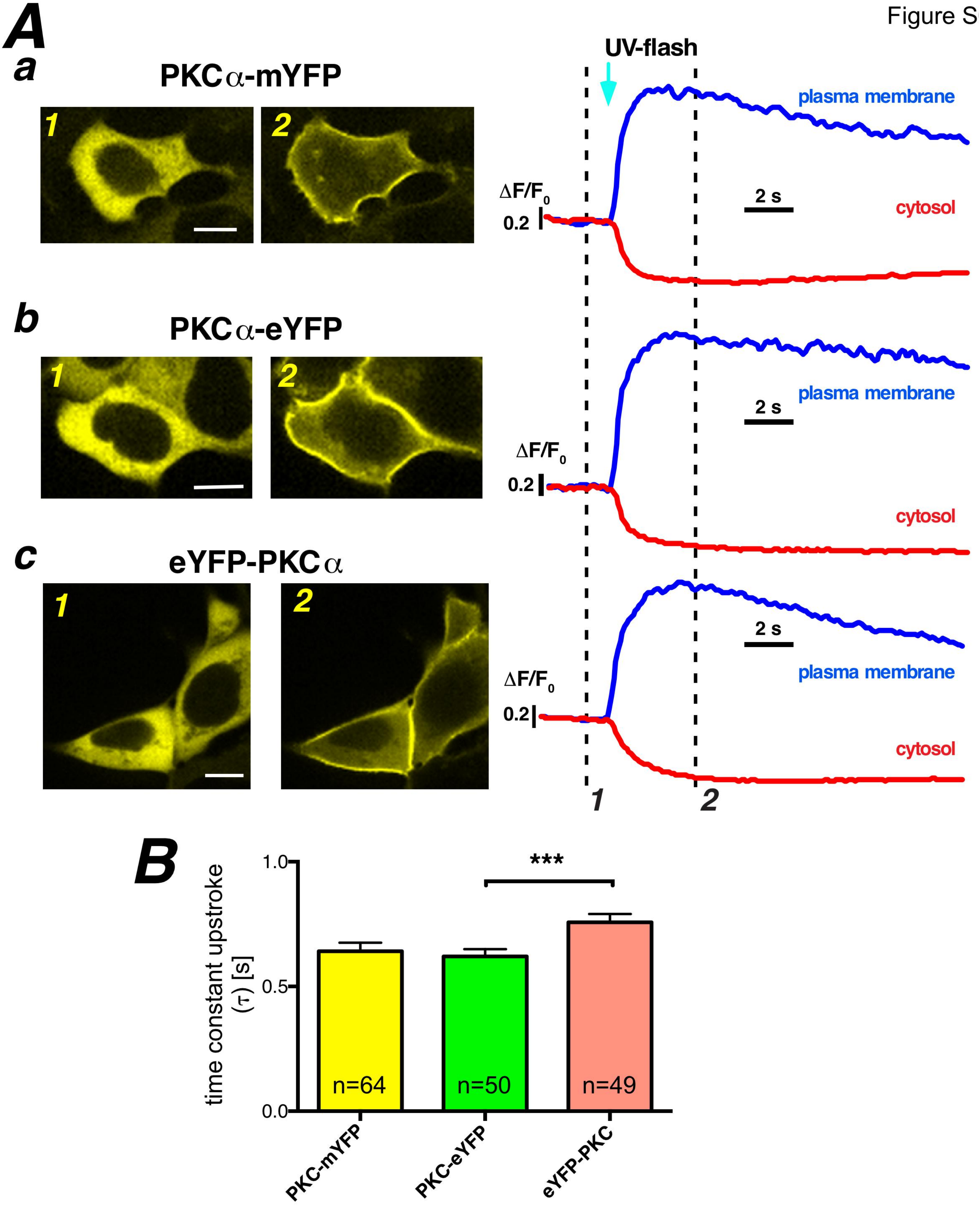

Figure S02. UV-flash evoked redestribution of PKCα-mYFP (Aa, B-yellow bar), PKCα-eYFP (Ab, B-green bar) and eYFP-PKCα (Ac, B-red bar). In (A) left panels depict exemplified confocal sections at the time points highlighted in the fluorescence over time traces in the right panels.
